# Age-Related Hearing Loss and Cognitive Decline: You Haven't Heard the Half of It

**DOI:** 10.3389/fnagi.2017.00112

**Published:** 2017-04-25

**Authors:** Dale Hewitt

**Affiliations:** Audiology, Portsmouth Hospitals NHS Trust, Queen Alexandra HospitalPortsmouth, UK

**Keywords:** age-related hearing loss, brain plasticity, cognitive decline, cross-modal cortical re-organization, hearing aids, listening effort, retirement

It has long been accepted that without hearing aid rehabilitation those with severe or greater hearing losses (thresholds above 70 dB HL) find conversation particularly difficult. That this in turn can lead to feelings of isolation, a reduced quality of life, and in some cases depression (Fortunato et al., 2016). This cascade of conditions can result with young and old adults alike. With mild to moderate sloping age-related hearing loss (thresholds up 70 dB HL), then not wearing hearing aids does not seem to trigger any comparable cascade.

Healthy aging is associated with neurophysiological and microvascular changes and this typically means the simultaneous onset of age-related sensory loss (including age-related hearing loss) and cognitive decline. Cross-sectional studies provide strong evidence for this link - see Roberts and Allen (2016) for a review. A study by Humes et al. (2013) showed a significant correlation between global sensory processing and global cognitive processing that was independent of age. Only relatively recently has serious attention being given to the idea that there might be a causal relationship between hearing loss and cognitive decline. Lin (2011) found that those with mild to moderate age-related hearing loss (MARHL) scored lower than those with normal hearing on the Digit Symbol Substitution Test - a nonverbal test that assesses executive function and psychomotor processing. Lin et al. (2013) found that, compared to those with normal hearing, those with untreated MARHL risk accelerated cognitive decline. Peripheral hearing was shown to be significantly related to measures of processing speed, memory, and global cognitive status in a study by Harrison Bush et al. (2015). Similar results are also emerging from the field of Gerontology (see Mosca and Wright, 2016; Tumino, 2016), with studies concluding that cognitive decline is associated with retirement from work. As the use of our cognitive skills becomes more limited, does this then hasten their decline?

Many individuals with MARHL seem to manage without hearing aids. Such a loss for someone whose employment involves demanding auditory tasks will most likely result in recourse to hearing aids. But a MARHL for someone who is retired from work and is happy to avoid any difficult listening environments, is far less likely to visit the audiology clinic. This is sometimes referred to by Audiologists as someone who has “enough hearing for their needs.”

But can hearing loss, as Lin (2011) suggests, precipitate cognitive decline? Can the use of hearing aids prevent cognitive decline? These questions have become the focus of recent investigations, prompted in part by the ever increasing numbers of people being diagnosed with dementia.

One particular focus of these recent investigations has concerned listening effort. Many studies have now shown that high intelligibility can still be achieved with speech degraded as a consequence of MARHL. But only at the cost of effort (and associated fatigue), through increased use of executive function and working memory. Reviews of many of these studies can be found in Hornsby et al. (2016), Peelle and Wingfield (2016), and Wayne and Johnsrude (2015). When listening to what others say in places with background noise, such as open-plan offices and restaurants, those with MARHL have to mentally work harder than those with normal hearing and seem to remember less of what was said. In cognitively normal older adults recent Gerontological studies show that deficits in everyday memory and executive function are strong predictors of the subsequent development of mild cognitive impairment (see Farias et al., 2017).

What of those who choose to withdraw from noisy situations, choose not to use (or have no access to) hearing aids, and consider themselves to have “enough hearing for their needs?” Recent studies involving the use of hearing aids and more established research findings involving the users of cochlear implants (CI) may help with our understanding. These studies describe the cross-modal cortical re-organization consequences of all types of hearing loss, including MARHL.

Neuroimaging studies have shown that peripheral hearing loss can result in both structural and functional changes in the auditory cortex. See Anderson et al. (2016) for a review. These changes include reduced gray matter volume and, as a consequence of cross-modal plasticity when auditory inputs are reduced, superior temporal brain regions becoming responsive to visual cues. Several studies (including Sandmann et al., 2012) propose that such deafness-related plasticity is maladaptive for auditory speech processing abilities when auditory input is later restored through the use of CI.

Sharma and Glick (2016) provide another review of the evidence for cross-modal cortical re-organization resulting from hearing loss. Here, is evidence that even those with MARHL demonstrate the recruitment of auditory cortex regions for visual stimulus. Auditory brain plasticity does not seem to require a prolonged period of deafness or for hearing loss to become severe before visual input begins to be used to supplement degraded auditory input.

To sum up so far. Common causes such as neural and microvascular aging are likely responsible for simultaneous cognitive decline and age-related hearing loss. However, the onset of MARHL seems to trigger its own cascade of conditions including the need for increased cognitive effort when attending to speech in noisy places, and brain plasticity initiating cross-modal auditory cortical re-organization.

Our attention needs then to turn toward understanding how and when hearing aids can be used to prevent cognitive decline. To what extent can hearing aids be used to reverse auditory system aging?

Initial results from this endeavor are encouraging. Doherty and Desjardins (2015) demonstrated that using hearing aids in the early stages of age-related hearing loss improved performance on auditory working memory tests in quiet and in background noise. Lavie et al. (2015) provide data which supports the hypothesis that hearing aid use can rehabilitate high-order central functions of the auditory system. Desjardins (2016) reported significant improvements in performance on cognitive test measures with hearing aid use, and then cognitive performance scores returning to baseline levels when hearing aids stopped being used. Qian et al. (2016) showed hearing aid use enabled higher scores on a test involving working memory.

We should not be surprised in the least that hearing aid use is able to reverse central auditory system aging. After all we knew already that the human auditory system has an inherent plasticity, and that this plasticity seems reasonably resistant to healthy aging. It is a plasticity that enables the auditory system to be trained (through experience) to undertake complex signal processing, to be fine-tuned to robustly encode a tonal language or a non-tonal language, and to separate speech from noise. It is a plasticity that enables cochlear implants to have successful outcomes for young and old alike. And last, but not least, a plasticity that will mean that speech recognition will become increasingly reliant upon visual input as the quality of the acoustic input slowly begins to reduce. See both Kraus and White-Schwoch (2015) and Lehmann and Skoe (2015) for considerably more about central auditory system plasticity.

We seem to have reached a conclusion about what should be done at the very onset of age-related hearing loss. It seems that without hearing aid rehabilitation a cascade of conditions will soon begin. And if this turns out to be true then it seems reasonable to hope that hearing aid rehabilitation with modern open-fit digital hearing aids can be used to postpone this cascade for several years at least. We await the results of the many current studies investigating the relationship between Age-Related Hearing Loss and Cognitive Decline. Of particular interest is a more thorough understanding of the triggering of cross-modal cortical re-organization resulting from hearing loss.

## Author contributions

The author confirms being the sole contributor of this work and approved it for publication.

### Conflict of interest statement

The author works in an NHS England Audiology department. NHS audiologists are employed to assess patients with hearing loss and to fit appropriate hearing aids - this includes the fitting of hearing aids to patients with a mild to moderate age-related hearing loss.
